# EEG Oscillatory Phase-Dependent Markers of Corticospinal Excitability in the Resting Brain

**DOI:** 10.1155/2014/936096

**Published:** 2014-06-11

**Authors:** Barbara Berger, Tamas Minarik, Gianpiero Liuzzi, Friedhelm C. Hummel, Paul Sauseng

**Affiliations:** ^1^Brain and Behaviour Research Group, School of Psychology, University of Surrey, Guildford, Surrey, GU2 7XH, UK; ^2^Clinic for Neurology, Universitätsspital Zürich, Raemistr. 100, 8091 Zürich, Switzerland; ^3^Brain Imaging and Neurostimulation Lab, Department of Neurology, University Medical Center Hamburg-Eppendorf, Martinistr. 52, 20246 Hamburg, Germany

## Abstract

Functional meaning of oscillatory brain activity in various frequency bands in the human electroencephalogram (EEG) is increasingly researched. While most research focuses on event-related changes of brain activity in response to external events there is also increasing interest in internal brain states influencing information processing. Several studies suggest amplitude changes of EEG oscillatory activity selectively influencing cortical excitability, and more recently it was shown that phase of EEG activity (instantaneous phase) conveys additional meaning. Here we review this field with many conflicting findings and further investigate whether corticospinal excitability in the resting brain is dependent on a specific spontaneously occurring brain state reflected by amplitude and instantaneous phase of EEG oscillations. We applied single pulse transcranial magnetic stimulation (TMS) over the left sensorimotor cortex, while simultaneously recording ongoing oscillatory activity with EEG. Results indicate that brain oscillations reflect rapid, spontaneous fluctuations of cortical excitability. Instantaneous phase but not amplitude of oscillations at various frequency bands at stimulation site at the time of TMS-pulse is indicative for brain states associated with different levels of excitability (defined by size of the elicited motor evoked potential). These results are further evidence that ongoing brain oscillations directly influence neural excitability which puts further emphasis on their role in orchestrating neuronal firing in the brain.

## 1. Introduction

Spontaneously occurring oscillatory electrical activity in the central and the peripheral nervous system reflects rhythmic changes in membrane potential. Thus, it was suggested that the phase of these oscillatory fluctuations of membrane potential is related to neural spiking [1]. Furthermore, the phase of a dominant rhythmical electroencephalographic (EEG) activity recorded in healthy humans is shown to influence response times to visual stimuli [2, 3]. This leads to the assumption that brain oscillations reflect fast cycles of cortical excitability changes and highlights the importance of spontaneous cortical oscillatory activity for stimulus perception and signal transmission.

Much of the past research in the area of human cortical oscillations looked at event-related amplitude decrease and increase. It has been shown that cortical deactivation and activation in various cognitive tasks involving different sensory modalities are selectively indicated by such amplitude changes (for an overview see [4]). These parameters have only a temporal precision of a few hundred milliseconds though, but as cortical neuronal communication happens in the range of only a few milliseconds EEG amplitude seems to rather reflect sustained activation and deactivation patterns [5]. To overcome this temporal restriction researchers have started to analyse instantaneous phase of brain oscillations in association with neural activity (e.g., [6]). Using EEG, the relation of ongoing oscillatory activity in the human brain in different frequency bands has been studied extensively during the last few years. It has been shown that these measures can be informative about a broad range of cognitive functions.

For instance, Hanslmayr and colleagues [7] conducted an EEG experiment in which participants had to distinguish between four different target letters (p, q, b, d) presented for only 57 ms. They were able to show that participants with high stimulus detection rate exhibited significantly less parieto-occipital prestimulus alpha (8–12 Hz) amplitude than participants with low stimulus detection rate. Furthermore, the more prestimulus alpha amplitude the less likely a stimulus was perceived. Likewise, Mathewson and colleagues [3] found that posterior prestimulus alpha amplitude is inversely correlated with stimulus detection in a metacontrast masking paradigm.

Neural oscillations dependent modulation of excitability can be studied even more convincingly when electrophysiological recordings are combined with neurostimulation techniques such as transcranial magnetic stimulation (TMS) as this, in contrast to correlative methods like EEG, makes it possible to prove causality. Romei and colleagues [8] used TMS to investigate neural excitability in the visual cortex. They induced illusory visual percepts (phosphenes) in blindfolded participants by applying TMS to the occipital pole of the cortex. By simultaneously recording EEG activity they were able to show that whether a phosphene was perceived or not was dependent on prestimulus alpha band amplitude at posterior brain areas. High prestimulus alpha amplitude resulted in failure to perceive the visual percept whereas low prestimulus alpha amplitude was associated with high visual cortex excitability and it was more likely that the phosphene would be perceived by the participant.

In a study similar to Romei and colleagues [8], but in the motor domain, Sauseng and colleagues [9] found that the likelihood of eliciting a motor evoked potential (MEP) by stimulating the primary motor cortex depends on the magnitude of prestimulus alpha band amplitude in the motor areas near the representation of the targeted muscle: the higher the prestimulus alpha band amplitude the lower the likelihood of eliciting an MEP. The authors conclude that the magnitude of corticospinal excitability is determined by the amount of topographically specific alpha oscillations in the sensorimotor cortex. Additionally, Zarkowski and colleagues [10] were able to show that not only alpha band activity is a determinant of whether TMS elicits an MEP or not but also other frequency bands such as gamma. They found a highly significant relation between prestimulus gamma band activity and elicited MEP amplitude size. Such studies illustrate that corticospinal excitability seems to be determined at least partly by the amplitude of spontaneously occurring ongoing cortical oscillations. But they still do not answer the question whether the phase of an ongoing oscillation is of relevance too.

This question was targeted by Busch et al. [11] who conducted an experiment in which they presented their participants with brief flashes of light (6 ms) at individual luminance threshold resulting in a detection rate of around 50% for all the subjects. They analysed the recorded EEG activity for perceived and non-perceived stimuli individually and found that the perception of the visual stimulus depended on the instantaneous phase of the theta (4–8 Hz) and alpha (8–12 Hz) frequency during the exact time of stimulus presentation and therefore provide strong evidence that visual perception is dependent on spontaneous oscillatory cortical activity. Analogously, in the motor domain van Elswijk and colleagues [12] presented data suggesting gain modulation of motor evoked potentials being related to instantaneous phase of corticospinal beta oscillations (around 20 Hz). Their participants had to abduct their index finger at 15% of maximal force while single TMS pulses were delivered to the contralateral primary motor cortex. The electromyogram (EMG) from the first dorsal interosseous (FDI) muscle and EEG from sensorimotor regions were recorded continuously. Instantaneous phase and amplitude at TMS application times were assessed offline for EMG and EEG. The main finding was that the amplitude of the MEP elicited by TMS was dependent on instantaneous beta phase of the EMG. van Elswijk and colleagues [12] could show very convincingly that a single TMS pulse delivered to the motor cortex during the rising phase of EMG beta frequency elicited maximal MEPs whereas TMS pulses applied during the falling phase of EMG beta activity produced smallest MEPs. Surprisingly, TMS-related muscle responses were associated neither with amplitude of ongoing EMG activity nor with instantaneous phase or amplitude of ongoing EEG activity. Ongoing EMG activity is strongly related to spinal neural activity but only weakly associated with EEG reflecting cortical activity from sensorimotor regions. Moreover, amplitude of the MEP does not only reflect cortical excitability but also excitability of spinal neurons. Given that in the reported study there was only an association found between EMG beta phase and MEP size but not any relation between ongoing EEG activity and MEP amplitude, van Elswijk and coworkers [12] suggested that the beta phase of spinal neurons at which they receive synaptic input is relevant for gain modulation of the resulting muscle response.

These results are in agreement with findings by Mitchell and colleagues [13] suggesting that most of the variance of a TMS-related muscle response is determined by EMG phase but, in contrast to van Elswijk et al. [12], also by EMG amplitude at a very broad frequency range comprising among others beta band frequency. What Mitchell and coworkers [13] also reported was a weak but significant association between EEG beta/gamma phase and amplitude explaining a small part of the variance of MEP amplitude size.

Another recent study by Mäki and Ilmoniemi [14] found a stronger association between EEG beta activity and MEP size when electroencephalographic measures were combined with single trial TMS. High sensorimotor EEG beta activity around 20 Hz was associated with low corticospinal excitability as reflected by small MEPs. Moreover, MEP size depended on instantaneous EEG beta phase at the time of stimulation. Interestingly, a beta phase dependence of MEPs was only obtained for EEG activity recorded over occipital and surprisingly not over sensorimotor sites. Taken together it is puzzling why these very similar studies report so contradictory findings with one study only documenting a relation between MEP size and EMG beta phase [12], one suggesting a relation between corticospinal excitability and broadband EMG amplitude and instantaneous phase as well as a weak association with sensorimotor EEG beta/gamma phase and amplitude [13], and Mäki and Ilmoniemi [14] who found a clear correlation between MEP size and sensorimotor EEG beta amplitude but also occipital beta phase (ongoing EMG activity had not been investigated in this study).

Importantly, EEG beta phase is in principle independent of amplitude. However, if it is considered that the signal recorded with EEG always consists of real neuronal activity plus a proportion of constant noise, and if one further considers that when the motor system is active and one obtains attenuated beta activity this can only be attributed to amplitude decrease of the neural signal whereas the level of noise will remain unchanged, it becomes clear that the relative proportion of noise in the recorded signal during suppressed beta activity is larger than during increased beta activity. Therefore, since EEG phase estimation is very sensitive to noise, instantaneous EEG beta phase will be more reliable when amplitude is high (e.g., during rest) than when beta is suppressed (i.e., during motor activity), highlighting the importance of studying the motor system during rest to be able to reliably detect basic mechanisms. This could explain why van Elswijk et al. [12] did not find any relation between EEG beta phase and MEP size but only an association between EMG beta phase (where beta amplitude was dominant) and MEP gain modulation. Mäki and Ilmoniemi [14] on the other hand reported a correlation between occipital EEG beta phase and MEP size. It is important to consider that during rest mainly EEG alpha activity but also beta activity is particularly high at posterior recording sites. Therefore, Mäki and Ilmoniemi's [14] positive finding might be related to a high signal to noise ratio for phase estimation at occipital recording sites. Independent of whether cortical oscillatory activity is associated with MEP gain modulation, the findings reported by van Elswijk et al. [12] and Mitchell et al. [13] suggesting spinal beta phase to modulate excitability seem to be very robust. Moreover, they suggest that ongoing oscillation phase-dependent excitability changes may be a very general mechanism found not only in the cortex but even in the spinal cord.

In order to be able to identify a general mechanism, independent of cognitive state of the brain and modality, it is therefore important to look at cortical activity beyond active information processing, general excitability during a resting state. Furthermore, given that findings regarding corticospinal excitability seem to be found predominantly but not at all exclusively in the beta frequency range (e.g., [10, 13]), it is important not to single this frequency band out but to look at the whole spectrum of frequencies.

Considering the importance of investigating associations of amplitude and instantaneous phase and corticospinal excitability during unconstrained rest, we present further evidence by investigating the association directly by applying TMS to the resting brain. We then correlated amplitude and instantaneous phase of EEG oscillations of different frequency bands within the primary motor cortex with MEP size. This should help to shed further light on the state which enables high corticospinal excitability in a resting brain and, therefore, optimal signal transmission/processing.

## 2. Materials and Methods

### 2.1. Participants

Ten healthy volunteers (4 females) took part in the experiment after giving written informed consent. Their mean age was 24.1 (range: 18 to 37 years) and all of them were right handed. The study was carried out in agreement with The Declaration of Helsinki (World Medical Association, 1996), and it was approved by the local ethics review committee.

### 2.2. EEG Acquisition

For EEG data acquisition 29 Ag-AgCl electrodes were mounted in an EasyCap (FMS) according to the extended 10–20-international system of electrode positions. Electrode adaptors near the TMS stimulation site were removed and the electrodes were taped to the electrode cap in order to keep the distance between TMS coil and scalp to a minimum. The electromyogram (EMG) was recorded from the first dorsal interosseous muscle (FDI) of the right hand. A digitally linked earlobe reference was used. Data were recorded at a sampling rate of 2000 Hz between DC and 70 Hz using a SynAmps (Neuroscan) amplifier and impedance was kept below 5 kΩ.

### 2.3. TMS Protocol

For applying the transcranial magnetic stimulation a Magstim 200 stimulator and a figure-of-eight shaped 70 mm coil (monophasic pulses with posterior-anterior current direction) were used.

The exact cortical representation of the FDI muscle was established for each participant individually by moving the TMS coil systematically within a grid of 2 mm over the left motor area (contralateral to the recorded FDI muscle) with the handle of the coil pointing backwards and laterally by approximately 30–45 degrees in respect to the sagittal midline. The individual resting motor threshold (rMT) was then determined. The rMT is defined as the output intensity of the stimulator that produces motor evoked potentials (MEPs) of more than 50 *μ*V in five out of ten consecutive trials [15]. For the volunteers in this experiment this was on average 49.3% of the maximal stimulator output. The individual rMT stimulation intensity was then used in the experiment.

### 2.4. Experimental Procedure

Volunteers were seated in a comfortable chair for the duration of the experiment with their right hand resting on a pillow. They were instructed to keep their eyes open and their hand relaxed. EEG was recorded continuously and single TMS pulses were delivered to the area of the left primary motor cortex representing the right FDI at intervals jittering between 4000 and 6000 ms to eliminate pulse anticipation. A total of 300 trials were run for each participant. As individual rMT was used for stimulation output this resulted in approximately half of the trials having an MEP below 50 *μ*V (conventionally considered as “subthreshold,” see, e.g., [15] for further information) and the other half above 50 *μ*V (“suprathreshold”).

### 2.5. EEG/EMG Data Analysis

Obtained EEG data were analysed offline using Neuroscan Edit (Neuroscan), BrainVision Analyzer software (BrainProducts), and Matlab (MathWorks). The sampling rate was changed to 2048 Hz. Data were then segmented into intervals from 500 ms prior to stimulation onset until stimulation onset. The individual segments were then visually inspected for muscle artefacts and eye blinks. Due to either such artefacts in the scalp EEG or the EMG showing tension in the FDI muscle prior to TMS pulse delivery an average of approximately 30% of the trials had to be discarded for every participant.

**Figure 1:**
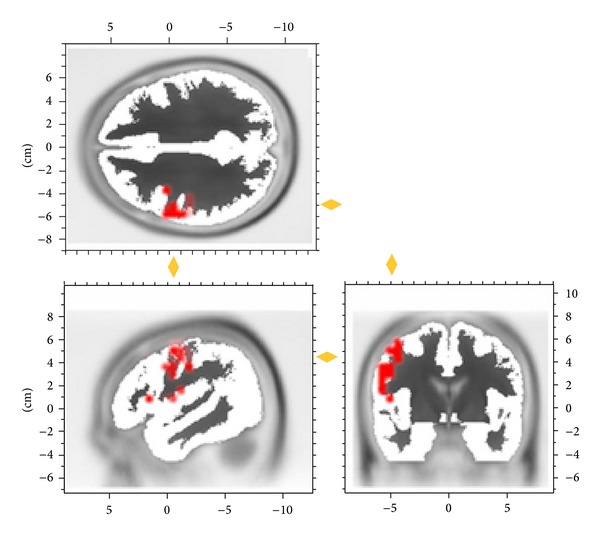
EEG amplitude and phase were extracted for an estimate of left primary motor cortex current source density. The a priori source for this activity is indicated in red.

The left precentral gyrus was offline defined as region of interest (ROI; see Figure 1). Using the LORETA algorithm (for further details see [16]) as implemented in Brain Vision Analyzer 2.0 current source density estimates from the left precentral gyrus were extracted for each single trial. Then, a continuous five-cycle complex Morlet wavelet transformation in steps of 4 Hz ranging from 6 to 70 Hz was applied to single trial current source density traces to obtain instantaneous amplitude and instantaneous phase for the time window of 500 ms prestimulation until stimulation onset. The EEG analyses were performed for six selected frequency bands' centre frequencies (theta 6 Hz, alpha 10 Hz, slow beta 18 Hz, fast beta 26 Hz, slow gamma 42 Hz, and fast gamma 62 Hz).

Finally, MEP peak-to-peak amplitude in a time-window of 20–40 ms poststimulus was obtained on a single trial basis. This allowed us to assess whether amplitude and instantaneous phase of EEG oscillatory activity were associated with MEP amplitude size.

### 2.6. Statistical Analysis

#### 2.6.1. Relation between Amplitude of EEG Oscillations and MEP Size

In order to explore whether the size of the amplitude of EEG oscillatory activity in the motor cortex has an effect on the size of the elicited MEP, Pearson's correlations (ranging from −1 to 1) between EEG amplitude size and the peak-to-peak amplitude of the elicited MEPs were calculated on single trial bases for each subject separately and for each sampling point within 500 ms before the TMS pulse.

For each participant and each frequency band individually, trials were then shuffled 500 times and correlations between EEG amplitude sizes and MEP size were calculated for these randomizations. The obtained coefficients purely based on chance were then set to an absolute value (between 0 and 1) before they were averaged resulting in an upper threshold. A lower threshold was obtained by multiplication of the absolute correlation values with −1 before calculating the average. Lastly, individual *t*-tests were used to compare the real correlation coefficients with the surrogate ones forming the upper and lower thresholds; this was done for each frequency band and each sampling point individually. For the amplitude of EEG oscillatory activity to significantly predict MEP size on a single trial basis, at least 10 consecutive correlation coefficients [17] of the real data need to be either significantly higher than the surrogate upper threshold correlation coefficients or significantly lower than the surrogate lower threshold correlation coefficients (*P* < 0.025, one-tailed).

#### 2.6.2. Relation between Instantaneous Phase and MEP Size

To investigate whether instantaneous phase prior to and at stimulation onset has an influence on MEP size and thus corticospinal excitability, circular linear correlations (for detailed information see [18]) were calculated between the instantaneous EEG phase angles and MEP peak-to-peak amplitude over all single trials (subthreshold as well as suprathreshold) for each subject separately. This was done for each sampling point within the 500 ms time window preceding the TMS pulse. This correlation coefficient can range between 0 and 1, with 0 indicating that instantaneous phase does not explain any variance of MEP size between trials; 1 indicates instantaneous phase angle at a given point in time being a perfect predictor for MEP amplitude size.

For each participant and each frequency band separately, the trials were then shuffled 500 times and circular linear correlations between instantaneous phase values and MEP size were calculated for each of these randomisations. Next, the averages of these 500 coefficients per sampling point purely based on chance were computed. Finally, *t*-tests comparing the real circular linear correlation coefficients with the surrogate coefficients were run on sample level for each sampling point and frequency band separately. Only data points where the real coefficient was significantly (*P* < 0.05, one-tailed) higher than the surrogate data were taken into consideration. A minimum of 10 consecutive *t*-tests [17] had to reach significance so that this was taken as indication that the instantaneous phase angle is significantly correlated with the size of the elicited MEP and therefore high corticospinal excitability. This criterion is considered as good procedure for multiple comparison correction in time series [17].

## 3. Results

### 3.1. MEP Size and EEG Amplitude

The size of the EEG amplitude of neither frequency band predicted the size of the elicited MEP. Real coefficients were always between the upper and the lower surrogate correlation coefficients which define the thresholds for significant results.

### 3.2. MEP Size and Instantaneous Phase

**Figure 2:**
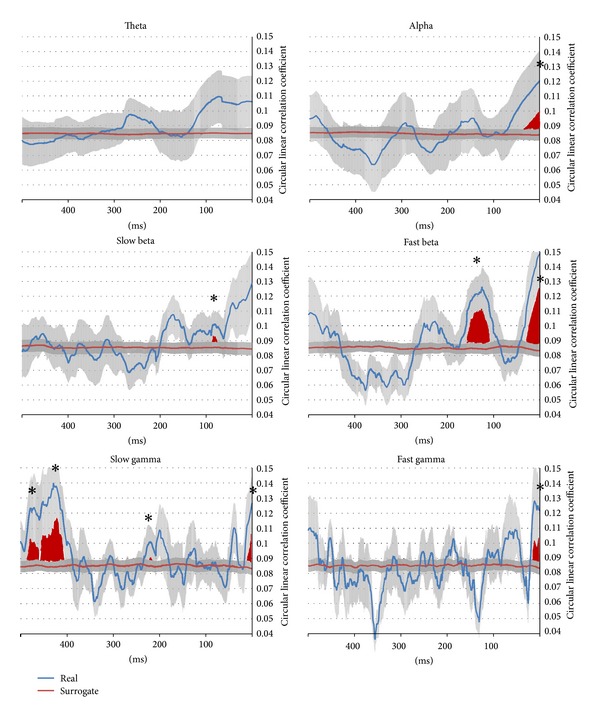
Temporal evolution of circular-linear correlation between instantaneous EEG phase and MEP size for 500 ms preceding TMS. The blue line indicates results from real data and the red line represents data based on surrogate data. Grey areas around the lines represent standard error of mean. Time windows where a significant difference between correlation coefficients based on real versus based on surrogate data was obtained for at least 10 successive sample points are indicated in red and with asterisks.

MEP size was predicted by instantaneous alpha, fast beta, and slow and fast gamma phase for the last 30 ms (alpha and beta) or 20 ms (gamma) before the time point of TMS application. Real circular-linear correlation coefficients were significantly higher than those based on surrogate data for at least 10 consecutive sampling points (*t* > 1.86, *P* < 0.05). Furthermore, instantaneous phase angles of slow and fast beta and slow gamma significantly correlated with the size of the elicited MEP at earlier time points (see Figure 2): for slow beta around 100 ms prior to TMS onset, fast beta between 150 and 100 ms before TMS, and for slow gamma effects were found between 500 and 400 ms and around 250 ms before TMS was delivered. Noteworthy is also that theta phase in the left primary motor cortex did not predict MEP size at all, nor did slow beta phase immediately before or at TMS onset.

## 4. Discussion

The present paper set out to investigate the role of the instantaneous oscillatory state of the resting brain associated with optimal corticospinal excitability and, therefore, signal processing and transmission. Additionally, we aimed to investigate this association further by applying single TMS pulses over the area in the left motor cortex representing the right FDI muscle, while simultaneously recording ongoing oscillatory activity with EEG.

### 4.1. Influence of Size of EEG Oscillatory Amplitude on Corticospinal Excitability

We found no correlations between the size of the elicited MEP and the size of the amplitude of EEG oscillatory activity in various different frequency bands. At first glance this might seem somewhat surprising as previous studies found such associations. Zarkowski and colleagues [10], for example, found a strong relation between prestimulus alpha and gamma amplitudes and elicited MEP amplitude size. Similarly, Sauseng et al. [9] report the strength of prestimulus alpha band amplitude predicting whether a TMS pulse would elicit an MEP or not, and Mäki and Ilmoniemi [14] found a strong correlation between MEP size and EEG beta amplitude. The striking difference between these studies and our results is that we investigated the time course of the amplitudes from 500 ms prestimulus until TMS pulse application whereas Zarkowski et al. [10] (as well as [9] and [14]) investigated the correlation by calculating a power spectrum over the whole pre-pulse time course. Considering that amplitude changes in the EEG are rather languid and have a huge variability over time it seems less surprising that we could not find significant associations. Zarkowski et al. [10] were able to obtain significant correlations because they investigated a longer time window of 2 seconds (which means overall more stability of amplitude) whereas we conducted our analysis for individual sampling points on single trial basis which makes the analysis strongly affected by variability. Furthermore, Sauseng and collegues [9] and Mäki and Ilmoniemi [14] compared two conditions - trials where the TMS pulse elicited a high MEP and trials that did not show an MEP response—and used the respective average for their analyses. Their results do not, strictly speaking, depict a correlation between the size of an MEP and ongoing EEG amplitude but rather an association of pre-TMS pulse EEG amplitude size and whether an MEP is elicited or not. Hence, the results are not directly comparable to the ones we describe here.

An explanation for the earlier described difference in findings regarding associations between EEG amplitude (mainly in the beta frequency band) and MEP gain modulation might be that some investigated the motor system during usage (grip force task) [12] whereas subjects in Mäki and Ilmoniemi's [14] experiment were in an unconstrained resting situation. Finger movements usually lead to strong amplitude decrease of sensorimotor EEG beta activity [19]. Therefore, when MEP gain modulation is investigated while the motor cortex is active (when EEG beta activity is suppressed) there will be less trial to trial variance of EEG beta amplitude due to a floor effect (beta amplitude is approaching zero). As a consequence, the chances that single trial instantaneous beta amplitude at stimulation time will successfully predict MEP variance are smaller when the active sensorimotor cortex is studied than during unconstrained rest (where there is higher beta amplitude obtained). This can explain why van Elswijk et al. [12] did not find a relation between cortical beta activity and MEP size whereas Mäki and Ilmoniemi [14] did. This argumentation is underlined by reports about a negative relation between EEG alpha amplitude recorded over sensorimotor cortex and MEP size [9, 10], since alpha amplitude exhibits similar dynamics of activation as beta frequency does.

### 4.2. Influence of Instantaneous Phase Angle on Corticospinal Excitability

The results from the circular-linear correlations indicate an association between instantaneous phase of oscillatory alpha, fast beta, and slow and fast gamma frequency band activity at left central sites (primary motor cortex, ROI) at the exact time of stimulation and size of the elicited MEP (a marker of corticospinal excitability). These results suggest that the motor system exhibits rapid fluctuations in excitability which seem to be determined by rhythmical fluctuations of electrical potential over left central recording sites at a broad range of frequencies.

Moreover, cortical excitability seems to be dependent on instantaneous phase of slow and fast beta and slow gamma at an earlier point in time (see Figure 2). Instantaneous phase of theta at primary motor areas seems not to be correlated with MEP size.

As highlighted in the introduction, a critical point is whether EEG phase influences corticospinal excitability or not, as findings have been controversial. We argue that our results make a strong case for neural excitability depending on oscillatory instantaneous phase by investigating spontaneous resting state EEG when rolandic rhythms are not suppressed in amplitude; that is, the relevant muscle is relaxed and the level of noise in the EEG does not diminish oscillatory activity.

In previous research [14] an association between occipital beta phase and MEP size was found. In the current study, however, instantaneous phase of alpha, fast beta, and gamma activity in sensorimotor cortex predicts MEP size. There are two arguments which might explain why in the current study phase at multiple frequency bands is related to corticospinal excitability: (i) Mäki and Ilmoniemi [14] sorted trials according to estimated phase. They analysed EEG on scalp level. Variation in dipole-orientation across participants might lead to some jitter of absolute phase on scalp level, particularly at central electrode sites, causing nil-findings of association between EEG phase and MEP size. In the current study EEG phase was estimated from source reconstructed signals. (ii) There might be variation in the preferred absolute instantaneous phase angle with maximal corticospinal excitability across participants. In contrast to previous work [14] we used circular-linear correlations between EEG phase and MEP size to analyse associations. Thus, interindividual differences in preferred phase angle did not negatively impact on the correlations.

### 4.3. EEG Phase Parameters as General Marker of Neural Excitability

Our findings suggest that very specific effects of instantaneous phase are associated with neural excitability. Whether the obtained pattern reflects a specific mechanism for corticospinal excitability or a general mechanism across different neural domains remains unclear and further investigations with stringent control conditions would be needed. Romei and colleagues [20] conducted a similar experiment in the visual domain in which they employed EEG and TMS for investigating cortical excitability in primary visual areas. They showed that the phase of instantaneous parieto-occipital alpha oscillations is crucial for whether a phosphene was elicited or not. They argue that the interpretation of alpha oscillations being associated with active inhibition mainly in task-irrelevant areas does only describe results regarding alpha amplitude and highlight the function of alpha oscillatory phase in situationally relevant cortical areas as controlling neural activation with high temporal precision (as discussed in [21]). This notion is supported by our present findings as we could find alpha phase dependent modulation of MEP size over the left motor cortex.

Evidence for oscillatory phase at stimulus onset influencing visual perception (and possibly excitability of visual brain areas) comes from a series of EEG studies. For instance, Mathewson and colleagues [3] associated perception of a masked visual stimulus with the phase of instantaneous alpha oscillations at posterior brain sites, and Busch and colleagues [11] obtained similar results in alpha but additionally in the theta frequency band.

All in all, it still needs to be seen whether instantaneous EEG phase as obtained in our study can be seen as a general mechanism determining neuronal excitability across modalities and neural systems. Very locally specific effects involving motor areas in the present study, as well as reported results from visual areas in experiments on visual perception, suggest very similar basic principles of rapid fluctuations of neural excitability implemented by spontaneous brain oscillations.

An important feature to point out is that instantaneous phase of a range of different frequencies seems to determine corticospinal excitability. It might be that an alignment of phases across frequencies, similar to a phenomenon described by Gruber et al. [22], needs to be achieved to obtain a brain state that is optimal for excitability.

At slow beta and slow gamma frequency, phase angle in relatively early time windows predicted corticospinal excitability. In the faster frequencies particularly it is unlikely that phase is highly stable over time. Therefore, these results are somewhat surprising. In particular beta band activity has been associated with top-down processing and the maintenance of a sensorimotor state [23]. It might be that input into primary motor cortex from hierarchically higher regions at beta and gamma frequency can lead to a change in excitability in subsequent, later time windows. This issue should be addressed by future research by investigating directed information transfer between motor areas and relating it to corticospinal excitability.

## 5. Conclusion

Here we have presented evidence suggesting that brain oscillations reflect spontaneous fluctuations of cortical excitability. This seems to be the case not only for instantaneous phase of oscillations in frequency bands dominant in the respective area (i.e., beta frequency in the motor domain) but also a wide range of various frequency bands around the stimulation site.

## References

[1] Lindsley DB (1952). Brain stem influences on spinal motor activity. *Research Publications: Association for Research in Nervous and Mental Disease*.

[2] Callaway E, Yeager CL (1960). Relationship between reaction time and electroencephalographic alpha phase. *Science*.

[3] Mathewson KE, Gratton G, Fabiani M, Beck DM, Ro T (2009). To see or not to see: prestimulus *α* phase predicts visual awareness. *The Journal of Neuroscience*.

[4] Klimesch W (1999). EEG alpha and theta oscillations reflect cognitive and memory performance: a review and analysis. *Brain Research. Brain Research Reviews*.

[5] Sauseng P, Klimesch W (2008). What does phase information of oscillatory brain activity tell us about cognitive processes?. *Neuroscience and Biobehavioral Reviews*.

[6] Buzsáki G, Draguhn A (2004). Neuronal olscillations in cortical networks. *Science*.

[7] Hanslmayr S, Aslan A, Staudigl T, Klimesch W, Herrmann CS, Bäuml K-H (2007). Prestimulus oscillations predict visual perception performance between and within subjects. *NeuroImage*.

[8] Romei V, Brodbeck V, Michel C, Amedi A, Pascual-Leone A, Thut G (2008). Spontaneous fluctuations in posterior *α*-band EEG activity reflect variability in excitability of human visual areas. *Cerebral Cortex*.

[9] Sauseng P, Klimesch W, Gerloff C, Hummel FC (2009). Spontaneous locally restricted EEG alpha activity determines cortical excitability in the motor cortex. *Neuropsychologia*.

[10] Zarkowski P, Shin CJ, Dang T, Russo J, Avery D (2006). EEG and the variance of motor evoked potential amplitude. *Clinical EEG and Neuroscience*.

[11] Busch NA, Dubois J, VanRullen R (2009). The phase of ongoing EEG oscillations predicts visual perception. *The Journal of Neuroscience*.

[12] van Elswijk G, Maij F, Schoffelen JM, Overeem S, Stegeman DF, Fries P (2010). Corticospinal beta-band synchronization entails rhythmic gain modulation. *The Journal of Neuroscience*.

[13] Mitchell WK, Baker MR, Baker SN (2007). Muscle responses to transcranial stimulation in man depend on background oscillatory activity. *The Journal of Physiology*.

[14] Mäki H, Ilmoniemi RJ (2010). EEG oscillations and magnetically evoked motor potentials reflect motor system excitability in overlapping neuronal populations. *Clinical Neurophysiology*.

[15] Rossini PM, Barker AT, Berardelli A (1994). Non-invasive electrical and magnetic stimulation of the brain, spinal cord and roots: basic principles and procedures for routine clinical application. Report of an IFCN committee. *Electroencephalography and Clinical Neurophysiology*.

[16] Pascual-Marqui RD, Michel CM, Lehmann D (1994). Low resolution electromagnetic tomography: a new method for localizing electrical activity in the brain. *International Journal of Psychophysiology*.

[17] Sander MC, Werkle-Bergner M, Lindenberger U (2011). Contralateral delay activity reveals life-span age differences in top-down modulation of working memory contents. *Cerebral Cortex*.

[18] Berens P (2009). CircStat: a matlab toolbox for circular statistics. *Journal of Statistical Software*.

[19] Pfurtscheller G (1981). Central beta rhythm during sensorimotor activities in man. *Electroencephalography and Clinical Neurophysiology*.

[20] Romei V, Gross J, Thut G (2012). Sounds reset rhythms of visual cortex and corresponding human visual perception. *Current Biology*.

[21] Sauseng P (2012). Brain oscillations: phase-locked EEG alpha controls perception. *Current Biology*.

[22] Gruber WR, Klimesch W, Sauseng P, Doppelmayr M (2005). Alpha phase synchronization predicts P1 end N1 latency and amplitude size. *Cerebral Cortex*.

[23] Engel AK, Fries P (2010). Beta-band oscillations—signalling the status quo?. *Current Opinion in Neurobiology*.

